# Real-World Evidence of Safety and Efficacy of Carboplatin plus Nanoparticle Albumin-Bound Paclitaxel in Patients with Advanced Non-Small-Cell Lung Cancer and Preexisting Interstitial Lung Disease: A Retrospective Study

**DOI:** 10.1155/2019/5315903

**Published:** 2019-03-20

**Authors:** Tomoyuki Araya, Toshiyuki Kita, Tsukasa Ueda, Nanao Terada, Tamami Sakai, Kenta Yamamura, Koji Kurokawa, Yuka Uchida, Takashi Sone, Hideharu Kimura, Kazuo Kasahara

**Affiliations:** ^1^Department of Respiratory Medicine, National Hospital Organization, Kanazawa Medical Center, 1-1 Shimoishibiki-Machi, Kanazawa 920-8650, Japan; ^2^Department of Respiratory Medicine, Cellular Transplantation Biology, Kanazawa University Graduate School of Medical Sciences, 13-1 Takara-Machi, Kanazawa 920-8641, Japan

## Abstract

**Background:**

Standard chemotherapy for advanced non-small-cell lung cancer (NSCLC) with preexisting interstitial lung disease (ILD) has not yet been established. Although a combination of carboplatin and paclitaxel is most frequently used for patients with advanced NSCLC and ILD, the safety and efficacy of carboplatin plus nanoparticle albumin-bound paclitaxel (nab-paclitaxel) are yet to be elucidated.

**Objectives:**

This study aimed to evaluate the safety and efficacy of carboplatin plus nab-paclitaxel for advanced NSCLC with ILD.

**Methods:**

This retrospective study included nine patients with advanced NSCLC and ILD who received carboplatin plus nab-paclitaxel as first-line chemotherapy at the National Hospital Organization Kanazawa Medical Center between April 2013 and December 2017. The ILD-GAP index was used to evaluate mortality risk of baseline ILD.

**Results:**

A usual interstitial pneumonia (UIP) pattern of ILD was observed in five (55.6%) patients on their baseline high-resolution computed tomography (HRCT) scans. The median ILD-GAP index was 4 (range, 1–5), and six (66.7%) patients had ILD-GAP index ≥4. We observed no ILD exacerbations or chemotherapy-related deaths. The overall response and disease control rates were 77.8% (95% CI, 40.0–97.2) and 88.9% (95% CI, 51.8–97.2), respectively. The median progression-free survival and overall survival were 5.8 months (95% CI, 2.1–7.7) and 8.0 months (95% CI, 2.6–16.8), respectively.

**Conclusions:**

Carboplatin plus nab-paclitaxel showed favorable safety and efficacy in patients who had advanced NSCLC and ILD with a high risk of mortality. Prospective studies are required to further confirm these results.

## 1. Introduction

Lung cancer is the leading cause of cancer-related mortality worldwide [1]. Interstitial lung disease (ILD), a critical problem in the treatment of lung cancer, is observed in 5.8–15.2% of patients with lung cancer at diagnosis [2, 3]. Although the treatment for non-small-cell lung cancer (NSCLC) has gradually improved in recent years [4], patients with advanced NSCLC and preexisting ILD have few therapeutic options. In addition, patients with advanced NSCLC with ILD have been excluded from clinical trials and, therefore, no standard chemotherapy regimen has been established for such patients. In daily clinical practice, cytotoxic chemotherapies are usually selected for the treatment of advanced NSCLC with ILD. However, cytotoxic chemotherapies can lead to acute exacerbation of ILD (AE-ILD) that is potentially fatal [5, 6]. Only two prospective single-arm studies of cytotoxic chemotherapies for lung cancer with ILD have been reported [7, 8]. Minegishi and colleagues [7] investigated the safety and efficacy of carboplatin plus weekly paclitaxel for the treatment of NSCLC with ILD. Patients received carboplatin (area under the curve (AUC) of 5) on day 1 and paclitaxel (100 mg/m^2^ on day 1, 8, and 15), every 4 weeks. In that study, only one patient (5.6%) experienced AE-ILD, the overall response rate (ORR) was 61%, and the median progression-free survival (PFS) and median overall survival (OS) were 5.3 and 10.6 months, respectively. Sekine and colleagues [8] investigated the safety and efficacy of carboplatin plus S-1 for the treatment of NSCLC with ILD. Patients received carboplatin (AUC = 5) on day 1 and S-1 (80 mg/m^2^, daily) for 14 days, every 3 weeks. In that study, two (9.5%) patients experienced AE-ILD, and the ORR was 33% while the median PFS and OS were 4.2 and 9.7 months, respectively. The combination of carboplatin and paclitaxel, which is the most frequently used regimen for advanced NSCLC worldwide [9, 10], is usually used for patients with NSCLC with ILD in Japan. The incidence rate of AE-ILD in patients administered this regimen was reported to be 0–27% [7, 11, 12], which was considered acceptable. Recently, a phase III study demonstrated that carboplatin plus nanoparticle albumin-bound paclitaxel (nab-paclitaxel) achieved a higher response rate than that of carboplatin plus paclitaxel without worsening the toxicity profile [13]. Importantly, the carboplatin plus nab-paclitaxel regimen did not increase the incidence of ILD compared to carboplatin plus paclitaxel. Therefore, carboplatin plus nab-paclitaxel is a promising regimen for advanced NSCLC with ILD. However, the safety and efficacy of carboplatin plus nab-paclitaxel in these patients are yet to be thoroughly investigated. The objective of this study was to evaluate the safety and efficacy of carboplatin plus nab-paclitaxel as first-line chemotherapy in patients with advanced NSCLC and preexisting ILD.

## 2. Patients and Methods

### 2.1. Patients

Medical records were retrospectively reviewed to collect data of consecutive patients with advanced NSCLC and ILD who received carboplatin plus nab-paclitaxel as first-line treatment between April 2013 and December 2017 at the National Hospital Organization Kanazawa Medical Center. This retrospective study was approved by the Institutional Review Board committee of the National Hospital Organization Kanazawa Medical Center (H30-008) and was conducted in accordance with the principles of the World Medical Association Declaration of Helsinki and Good Clinical Practice guidelines. All patients were selected by the physicians' judgment based on the following conditions: (i) histologically or cytologically confirmed nonresectable stage IIIB or stage IV NSCLC based on the 7th edition of the TNM classification for lung cancer [14], (ii) Eastern Cooperative Oncology Group (ECOG) performance status (PS) of 0–2, (iii) adequate organ function for systemic chemotherapy, and (iv) diagnosed with ILD. ILD was diagnosed based on clinical features and the findings of pretreatment chest high-resolution computed tomography (HRCT). Two radiologists and two pulmonologists evaluated the chest HRCT findings of our patients and classified ILD patterns according to the absence or presence of a typical usual interstitial pneumonia (UIP) pattern based on Fleischner Society White Paper and the ATS/ERS/JRS/ALAT 2018 [15, 16]. The ILD-GAP index, based on gender (G), age (A), and two lung physiology variables (P, forced vital capacity (FVC) and diffusing capacity of the lung for carbon monoxide (DLco)), which is a previously validated risk prediction method in patients with chronic interstitial lung disease [17], was used to evaluate the mortality risk of baseline ILD.

### 2.2. Treatment

Carboplatin was administered at a dose of AUC 5-6 on day 1, and nab-paclitaxel at a dose of 100 mg/m^2^ was administered on days 1, 8, and 15 every 4 weeks. Patients who experienced treatment-related adverse events (TRAEs) such as grade ≥3 neutropenia or thrombocytopenia received dose reduction or interruption at the physicians' discretion. Chemotherapy was repeated every 4 weeks for up to six cycles or until unacceptable toxicity occurred.

### 2.3. Evaluation of Response

The response to treatment was evaluated using the response evaluation criteria in solid tumors version 1.1 [18]. Chest and abdominal CT was performed every two cycles to evaluate the tumor size during chemotherapy.

### 2.4. Evaluation of AE-ILD and Other Toxicities

Toxicity was evaluated using the Common Terminology Criteria for Adverse Events, version 4.0. An AE-ILD was defined as an acute worsening or development of dyspnea within 4 weeks from the last day of chemotherapy, as well as CT findings showing new bilateral ground-glass opacification or consolidation superimposed on a background of interstitial shadow not fully explained by the presence of cardiac failure or fluid overload, or both [6].

### 2.5. Statistical Analysis

The primary endpoint of this study was evaluation of the incidence rate of AE-ILD with carboplatin plus nab-paclitaxel, and the secondary endpoints were evaluation of the ORR, PFS, OS, and toxicities other than AE-ILD with carboplatin plus nab-paclitaxel. PFS ad OS were assessed using the Kaplan–Meier method. PFS was defined as the period from day 1 of chemotherapy until disease progression or death from any cause or the last follow-up, whichever occurred first. OS was defined as the period from the start of chemotherapy until death from any cause. All statistical analyses were performed using the Statistical Package for the Social Sciences (SPSS) version 24.0 (IBM Corp., Armonk, NY, USA). *P* values < 0.05 were considered statistically significant.

## 3. Results

### 3.1. Patient Characteristics

**Figure 1:**
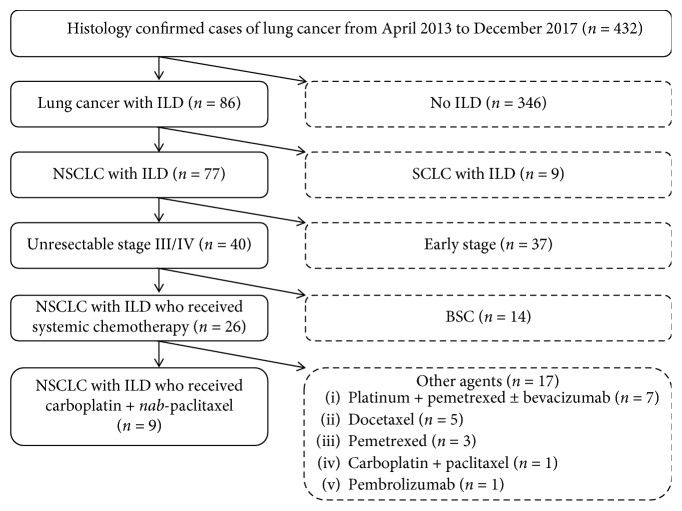
CONSORT diagram. ILD: interstitial lung disease; NSCLC: non-small-cell lung cancer; SCLC: small-cell lung cancer; BSC: best supportive care.

Of the 432 patients finally diagnosed with lung cancer from April 2013 to December 2017, 26 patients with advanced or recurrent NSCLC with ILD received systemic chemotherapy. These patients included nine who were treated with carboplatin plus nab-paclitaxel as first-line chemotherapy and were enrolled in this study. The remaining 17 patients with ILD received other anticancer agents (platinum plus pemetrexed with or without bevacizumab in seven, carboplatin plus paclitaxel in one, docetaxel monotherapy in five, pemetrexed monotherapy in three, and pembrolizumab in one), as shown in Figure 1.

The baseline patient characteristics are shown in Table 1. The median age of our patients was 68 (range, 59–78) years, and 88.9% (8/9) were men. All patients were current or former smokers, and 77.8% (7/9) had an ECOG PS of 0 or 1. Two and seven patients had stage IIIB and IV disease conditions, respectively. The histological subtypes mostly consisted of squamous cell carcinoma (*n* = 7), adenosquamous carcinoma, and pleomorphic carcinoma (*n* = 1 each). None of our patients had driver mutations including mutations of epidermal growth factor receptor or anaplastic lymphoma kinase translocations. UIP pattern of ILD was observed in five (55.6%) patients on their baseline HRCT scans. The results of the baseline pulmonary function tests are shown in Table 1. The median percentage predicted forced vital capacity (FVC), ratio of forced expiratory volume in 1 s (FEV1) to the FVC, and percentage predicted diffusing capacity of the lung for carbon monoxide (DLco) were 112.4% (range, 53.8–124.2%), 0.77 (range, 0.56–0.92), and 55.8% (range, 29.1–76.6%), respectively. Low FVC (%FVC < 75%) was observed in four (44.4%) patients, and DLco decline (%DLco < 55%) occurred in seven (77.8%) patients. The median ILD-GAP index was 4 (range, 1–5), and six (66.7%) patients had ILD-GAP index ≥4.

### 3.2. Treatment Exposure

The median number of treatment cycles was four (range, 1–6 cycles). The median cumulative nab-paclitaxel dose was 840 mg/m^2^ with the median relative dose intensity (RDI) of 70% (range, 32.1–90%). The median cumulative carboplatin dose was 1695 mg, with a median RDI of 70% (range, 49.5–90%). The frequency of dose reductions, interruptions, and delays of nab-paclitaxel was 66.7%, 33.3%, and 88.9%, respectively. The frequency of carboplatin dose reduction was 66.7%. These dose modifications were mainly due to neutropenia. There were no cases of monotherapy with nab-paclitaxel or treatment discontinuation due to intolerability of platinum doublet chemotherapy. None of our patients received antifibrotic agents such as pirfenidone or nintedanib during study treatment because of lack of evidence regarding combination therapy.

### 3.3. Incidence Rate of AE-ILD and Other Toxicities

TRAEs are summarized in Table 2. For hematological toxicities, grade 3 or 4 neutropenia, leukopenia, anemia, and thrombocytopenia were observed in four, three, five, and three (44.4, 33.3, 55.6, and 33.3%) patients, respectively. No cases of febrile neutropenia were observed. For nonhematological toxicities, no chemotherapy-induced AE-ILD or chemotherapy-related deaths were observed, whereas lung infections needing intravenous antibiotics were observed in three (33.3%) patients. Furthermore, grade ≥3 sensory neuropathies were not observed.

### 3.4. Efficacy

**Figure 2:**
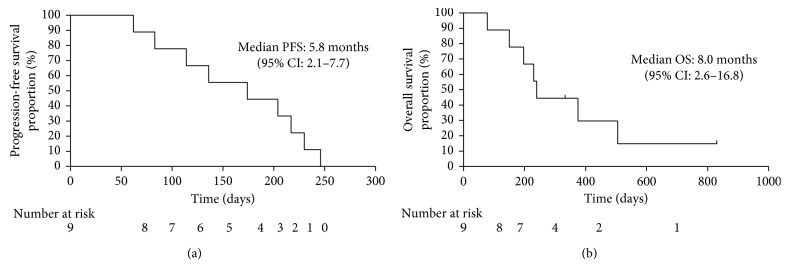
Kaplan–Meier curves of progression-free survival (a) and overall survival (b) in patients with advanced non-small-cell lung cancer (NSCLC) and interstitial lung disease treated with carboplatin plus nab-paclitaxel. OS: overall survival; PFS: progression-free survival; PS: performance status; CI: confidence interval.

Seven patients had partial response (PR), and one had stable disease (SD). The ORR and disease control rate were 77.8% (95% CI, 40.0–97.2) and 88.9% (95% CI, 51.8–97.2), respectively, as shown in Table 3. The median PFS and OS were 5.8 months (95% CI, 2.1–7.7) and 8.0 months (95% CI, 2.6–16.8), respectively. The Kaplan–Meier curves of the PFS and OS are shown in Figures 2(a) and 2(b), respectively.

### 3.5. Posttreatment

After disease progression was confirmed, six (66.7%) patients received second-line and later chemotherapy including docetaxel and nivolumab. Of these patients, AE-ILD developed in two patients (one of three patients treated with docetaxel and one of four patients treated with nivolumab). Both patients were successfully treated with corticosteroids.

## 4. Discussion

This study was conducting using data from a real-world setting, which demonstrated that carboplatin plus nab-paclitaxel was a feasible, well-tolerated, and effective chemotherapy regimen in patients with NSCLC with ILD. It is noteworthy that no chemotherapy-induced AE-ILD or chemotherapy-related deaths occurred in our patients, although two-thirds had high mortality risk (18.2% death in 1 year) estimated ILD-GAP index, a predictor of mortality in chronic ILD [17]. Reportedly, three retrospective studies indicated similar safety results of carboplatin plus nab-paclitaxel in patients with advanced NSCLC with ILD in first-line settings [19–21]. A summary of the characteristics of these three studies and the present study is shown in Table 4.

These studies indicated that the incidence rate of AE-ILD was 0–8.3%, which was similar to the 8.5% 1-year AE frequency of idiopathic pulmonary fibrosis (IPF) in the natural course [22] and was a very low frequency compared to that in previous studies [7, 8, 11, 12, 23, 24]. However, the severity of ILD was not sufficiently evaluated in three previous studies using carboplatin plus nab-paclitaxel [19–21]. Two of three studies evaluated only %FVC or percentage predicted vital capacity, and one study did not evaluate the baseline pulmonary function. Therefore, our study is the first to demonstrate the safety of carboplatin plus nab-paclitaxel in patients with advanced NSCLC with high mortality risk ILD, evaluated using the ILD-GAP as the standard risk assessment method. Based on the observed safety profile, we believe that carboplatin plus nab-paclitaxel could be selected as a first-line chemotherapy regimen in patients with advanced NSCLC with ILD because AE-ILD is the principal reason for shortened survival in such patients. Regarding the ILD pattern, it is important to evaluate the risk of acute exacerbation before the initiation of chemotherapy for NSCLC with ILD. Kenmotsu and colleagues [23] reported that the incidence rate of AE-ILD was significantly higher in patients with lung cancer with UIP pattern than it was in patients with non-UIP pattern (30% versus 8%). Nonetheless, none of the five patients who presented IPF patterns in our study experienced AE-ILD. Therefore, we believe that carboplatin plus nab-paclitaxel could be a safe treatment choice for patients with advanced NSCLC and preexisting ILD including IPF. Accumulating further real-world data regarding carboplatin plus nab-paclitaxel will assist building a rationale to conduct a large-scale randomized study of lung cancer with interstitial lung disease in the future.

The response rate and PFS of carboplatin plus nab-paclitaxel in this study were comparable with those in the CA031 study [13, 25]. Considering that our population mainly consisted of patients with squamous cell carcinoma, the OS in this study also was not shorter than that in the squamous cell carcinoma subgroup of the CA031 trial [26]. Similarly, previous retrospective studies using carboplatin plus nab-paclitaxel demonstrated good efficacy OS results of 8.1 to 14.9 months in patients with advanced NSCLC with ILD in a first-line setting [19–21]. Therefore, we believe that the efficacy of the carboplatin plus nab-paclitaxel in patients with advanced NSCLC with ILD might be equivalent to that in patients without ILD.

The incidence rate of hematological toxicities in this study was consistent with that in the Japanese subset of the CA031 trial [13, 25]. We think that this result indicated that ILD did not increase hematological toxicities. However, the incidence rate of grade ≥3 lung infection in this study was higher than that in CA031 trial. However, lung infections in our patients were successfully treated with intravenous antibiotics and consequently did not lead to the onset of AE-ILD whereas AE-ILD is reported to be associated with infections [27].

All our patients with ILD were diagnosed as having ILD at the same time of advanced NSCLC; therefore, none of our patients received antifibrotic agents such as nintedanib during treatment. However, there has been sufficient evidence to favor the use of the combination of nintedanib and cytotoxic agents. The LUME-Lung 1 and 2 phase III trials demonstrated that nintedanib at a dose of 200 mg twice daily on days 2–21 plus docetaxel at a dose of 75 mg/m^2^ on day 1 or pemetrexed at a dose of 500 mg/m^2^ on day 1 of each 21-day cycle resulted in a significantly longer progression-free survival, compared to docetaxel or pemetrexed alone, with a manageable safety profile in the second-line setting [28, 29]. Moreover, in the first-line setting, nintedanib demonstrated an acceptable safety profile in two phase I trials [30, 31]. Doebele and colleagues [30] reported that nintedanib at a dose of 200 mg twice daily on days 2–21 in association with paclitaxel at a dose of 200 mg/m^2^ and carboplatin at AUC 5 on day 1 of each 21-day cycle as first-line treatment for advanced NSCLC demonstrated acceptable safety. Forster and colleagues [31] reported that nintedanib at a dose of 200 mg twice daily on days 2–7 and 9–21 combined with cisplatin (75 mg/m^2^, day 1) plus gemcitabine (1250 mg/m^2^, days 1 and 8) of each 21-day cycle as first-line treatment for advanced squamous NSCLC demonstrated a manageable safety profile in the LUME-Lung 3 trial. Based on the findings of those trials, we believe that nintedanib in combination with cytotoxic chemotherapies can be a promising therapeutic approach for patients with advanced NSCLC and IPF. More recently, the J-SONIC trial (UMIN000026799), a phase III trial is ongoing for comparing the efficacy and safety of combination therapy of nintedanib at a dose of 150 mg twice daily and carboplatin at AUC 6 plus nab-paclitaxel at a dose of 100 mg/m^2^ on days 1, 8, and 15 of each 21-day cycle with those of carboplatin plus nab-paclitaxel alone [32].

Importantly, Tzouvelekis and colleagues [33] described that implementation of aggressive diagnostic and therapeutic strategies should generally be avoided in patients with IPF and lung cancer because the risk for complications including acute exacerbations (AEs) outweighs the benefits depending on IPF severity and the patient's performance status. Consistent with this report, diagnoses in all nine of our study patients were made using tumor samples obtained by bronchoscopic examination. Of those, diagnoses were made in 77.8% (7/9) patients using samples obtained by endobronchial ultrasound-guided transbronchial needle aspiration, which is a noninvasive diagnostic strategy. The same is true for therapeutic strategies. Even though carboplatin plus nab-paclitaxel is safe, we should set a minimum necessary requirement for systemic chemotherapy, including good performance status and absence of respiratory failure, in consultation with a multidisciplinary team.

There are a few limitations in this study. First, our study was retrospective and included a small sample size, which limited the statistical power of the analyses. Second, the diagnosis of preexisting ILD pattern was mainly based on the CT findings and not on histology. Therefore, the ILD pattern could not be exactly assessed. In addition, most of the patients received the best supportive care alone also in daily practice. Therefore, the safety of this combination regimen for NSCLC with severe ILD was undetermined. Third, the dose modifications of carboplatin or nab-paclitaxel were based on the discretion of the attending doctors, and therefore, not standardized among the patients.

## 5. Conclusions

Carboplatin plus nab-paclitaxel showed favorable safety and efficacy in patients who had advanced NSCLC and ILD with a high risk of mortality. Further studies with larger numbers of patients are required to confirm our results.

## Figures and Tables

**Table 1:** Baseline patient characteristics.

Age, years	
Median (range)	69 (59–79)
Gender	
Male	8
Female	1
Smoking status	
Current	3
Former	6
Preexisting interstitial lung disease	
IPF	5
Non-IPF	4
ECOG performance status score	
0-1	7
2	2
Disease stage	
IIIB	2
IV	7
Tumor histology	
Squamous cell carcinoma	7
Adenosquamous carcinoma	1
Pleomorphic carcinoma	1
EGFR mutation status	
Wild type	9
ALK fusion genes	
Negative	4
Unknown	5
KL-6 (U/mL)	
Median (range)	659 (317–3890)
SP-D (ng/mL)	
Median (range)	80.9 (43–174)
%FVC (%)	
Median (range)	112.4 (53.8–124.2)
FEV1/FVC (%)	
Median (range)	77.1 (56.4–92.4)
%DLco (%)	
Median (range)	55.8 (29.1–76.6)
ILD-GAP index	
Median (range)	4 (1–5)

IPF: idiopathic pulmonary fibrosis; ECOG: Eastern Cooperative Oncology Group; EGFR: epidermal growth factor receptor; ALK: anaplastic lymphoma kinase; FVC: forced vital capacity; FEV1: forced expiratory volume in one second; DLco: carbon monoxide diffusing capacity of the lung; ILD: interstitial lung disease; GAP: gender, age, and lung physiology variables.

**Table 2:** Toxicities.

Adverse events	All grades	Grade ≥3
	*n* (%)	*n* (%)
Hematological AEs		
Leukopenia	9 (100)	3 (33.3)
Neutropenia	9 (100)	4 (44.4)
Anemia	6 (66.7)	5 (55.6)
Thrombocytopenia	5 (55.6)	3 (33.3)
Febrile neutropenia	0 (0)	0 (0)
Nonhematologic AEs		
Alopecia	6 (66.7)	0 (0)
Anorexia	5 (55.6)	1 (11.1)
Lung infection	4 (44.4)	3 (33.3)
Fatigue	3 (33.3)	0 (0)
Sensory neuropathy	2 (22.2)	0 (0)
Nausea	1 (11.1)	0 (0)
Pneumonitis	0 (0)	0 (0)
Sepsis	0 (0)	0 (0)

AE: adverse event.

**Table 3:** Objective tumor response.

Response	No. of patients
Complete response	0
Partial response	7
Stable disease	1
Progressive disease	1
Response rate (%)	77.8
Disease control rate (%)	88.9

**Table 4:** Summary of characteristics of the present study and previous studies using carboplatin plus nab-paclitaxel in patients with advanced non-small-cell lung cancer and with interstitial lung disease in first-line settings.

Reference	No. of patients	Study patients	Study design	AE-ILD, *n* (%)	ORR (%)	Median PFS (months)	MST (months)
[19]	9	NSCLC with ILD	Retrospective	0 (0)	55.6	5.8	11.5
[20]	12	NSCLC with ILD	Retrospective	1 (8.3)	66.7	5.1	14.9
[21]	8	SCC with ILD	Retrospective	0 (0)	50.0	5.6	8.1
Present study	9	NSCLC with ILD	Retrospective	0 (0)	77.8	5.8	8.0

AE-ILD: acute exacerbation of interstitial lung disease; ORR: overall response rate; PFS: progression-free survival; MST: median survival time; NSCLC: non-small-cell lung cancer; SCC: squamous cell carcinoma.

## Data Availability

The data used to support the findings of this study are available from the corresponding author upon request.

## Abbreviations

ILD: Interstitial lung disease
NSCLC: Non-small-cell lung cancer
AE: Acute exacerbation
AUC: Area under the curve
ORR: Overall response rate
PFS: Progression-free survival
OS: Overall survival
ECOG: Eastern Cooperative Oncology Group
PS: Performance status
HRCT: High-resolution computed tomography
UIP: Usual interstitial pneumonia
GAP: Gender, age, and lung physiology variables
TRAE: Treatment-related adverse event
FVC: Forced vital capacity
DLco: Diffusing capacity of the lung for carbon monoxide
FEV1: Forced expiratory volume in 1 s
RDI: Relative dose intensity
PR: Partial response
SD: Stable disease
CI: Confidence interval
IPF: Idiopathic pulmonary fibrosis.
